# Adoption of the Coronary Artery Disease-reporting and Data System: Reduced Downstream Testing and Cardiology Referral Rates in Patients with Non-obstructive Coronary Artery Disease

**DOI:** 10.7759/cureus.5708

**Published:** 2019-09-20

**Authors:** Joshua Boster, Robert Hull, Michael U Williams, Jeremy Berger, Alec Sharp, Emilio Fentanes, Christopher Maroules, Ricardo Cury, Dustin Thomas

**Affiliations:** 1 Internal Medicine, Brooke Army Medical Center, Fort Sam Houston, USA; 2 Cardiology, Brooke Army Medical Center, Fort Sam Houston, USA; 3 Cardiology, Rhode Island Hospital, Warren Alpert Medical School of Brown University, Providence, USA; 4 Flight Medicine, Little Rock Air Force Base, Little Rock, USA; 5 Cardiology, Tripler Army Medical Center, Honolulu, USA; 6 Radiology, Naval Medical Center Portsmouth, Portsmouth, USA; 7 Radiology, Baptist Health South Florida, Miami, USA

**Keywords:** cad-rads, subspecialty referral, ccta, coronary ct angiography, downstream testing, cost

## Abstract

Introduction

The coronary artery disease-reporting and data system (CAD-RADS) was developed to standardize communication of per-patient maximal stenosis and provide treatment recommendations that may affect downstream testing.

Methods

Downstream testing, cardiology referral, and cost were abstracted for 1,796 consecutive patients undergoing coronary CT angiography (CCTA) before and after the adoption of the CAD-RADS reporting template at a single-center closed referral hospital system. Cost analysis was based on direct invasive and non-invasive testing utilizing the Center for Medicare & Medicaid Services (CMS) outpatient prospective payment system (OPPS) final rule for 2018.

Results

Baseline cardiovascular risk factors were balanced between the groups. Overall, referrals for downstream testing were similar between cohorts (10.7% vs 10.8%; *p* = 0.939). Referral for downstream testing was reduced in the CAD-RADS 1 & 2 cohort compared to non-obstructive coronary artery disease (CAD) by non-standardized reporting (NSR; 5.1% vs 14.4%, *p *< 0.001). This was offset by more non-diagnostic scans in the CAD-RADS cohort (9.7% vs 4.2%, *p *< 0.001), resulting in increased downstream testing (28.8% vs 11.4%, *p *= 0.038). Overall, cardiology referral rates by primary care providers (PCPs) were similar between the groups (12.2% vs 15.8%, *p *= 0.197). Cardiology referral rates were increased among patients with non-obstructive CAD in the NSR cohort compared with CAD-RADS 1 & 2 patients (20.5% vs 8.6%, *p *= 0.021). Referrals for invasive coronary angiography were low in both groups overall (3.5% vs 3.2%, *p *= 0.726). Median downstream testing costs were similar between the groups (*p *= 0.554).

Conclusions

Adoption of the CAD-RADS reporting template was associated with a reduction in downstream testing and cardiology referral rates among non-obstructive CAD (CAD-RADS 1 & 2) patients. Thus, CAD-RADS may impact downstream testing in patients in whom further testing can typically be deferred.

## Introduction

Coronary CT angiography (CCTA) is well-validated as an initial test in patients without known coronary artery disease (CAD) presenting with acute and stable chest pain [1-7]. The primary advantage of CCTA in this population is the ability to exclude significant CAD given its near 100% negative predictive value. Conversely, the inability to determine the functional significance of intermediate grade lesions has historically resulted in increased rates of referral for invasive coronary angiography (ICA) and revascularization. In the acute chest pain population, CCTA was observed to increase ICA rates revascularization rates compared with usual care [8]. Similarly, a trend toward increased ICA rates and significantly higher rates of revascularization following CCTA in the stable chest pain population were observed in a meta-analysis incorporating the four randomized controlled trials performed to date [9]. This data led to concern among payers and policymakers that CCTA may simply increase cost over functional testing. More recently, CCTA was adopted as the preferred first-line test in the National Institute for Health and Care Excellence (NICE) guidelines for the evaluation of typical and atypical angina in patients without known CAD in the United Kingdom (UK). This recommendation was based, in large part, on an analysis performed by independent economists evaluating the incremental cost and care benefit of various diagnostic strategies. They found that the cost of CCTA would have to triple before it would lose its cost-effectiveness advantage with respect to cost per correct diagnosis [10]. 

The role of the imaging report is vital to communicating pertinent test findings to the referring provider so that appropriate interventions can be pursued. The American College of Radiology (ACR) states that effective communication of important imaging findings should promote optimal patient care, support the ordering provider, be tailored to satisfy the need for timeliness, and minimize the risk of communication errors [11]. Data from the Study of Myocardial Perfusion and Coronary Anatomy’s Roles in Coronary Artery Disease (SPARC) demonstrated that referral for invasive coronary angiography was <10% and less than half of patients with the highest risk imaging findings were prescribed anti-anginal medications, aspirin, and lipid-lowering agents at 90 days following the index functional stress test or CCTA [12]. Coronary artery disease-reporting and data system (CAD-RADS™) was developed to bridge the communication gap between the cardiac CT imaging specialist and the ordering provider with a goal of concise disclosure of imaging results in a standardized fashion linked with recommended therapeutic interventions [13]. We sought to evaluate the impact of CAD-RADS adoption on downstream testing, referral for cardiology consultation, and direct costs in a single-center closed referral system.

## Materials and methods

Population

**Figure 1:**
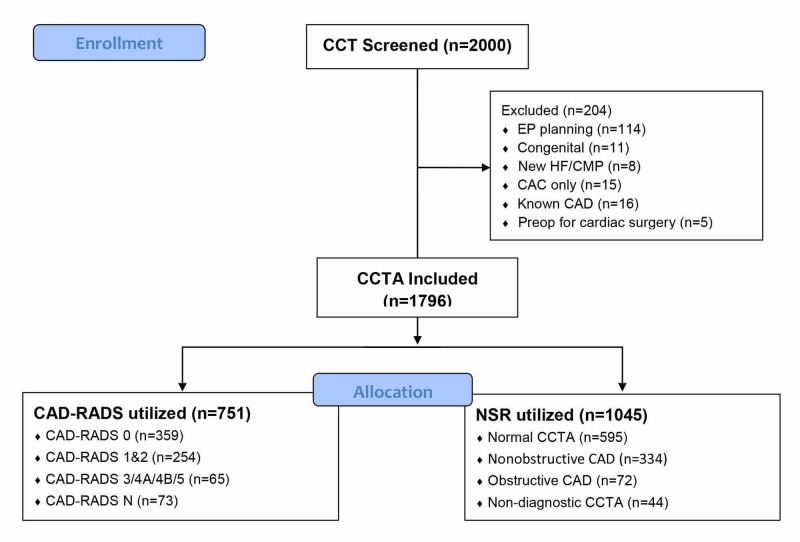
Study subject flow chart Cardiac computed tomography (CCT) scans for 2,000 total patients obtained before and after the adoption of CAD-RADS were screened. CCT scans performed for EP planning purposes, congenital heart disease, transcatheter aortic valve replacement planning, evaluation of new cardiomyopathy or heart failure, other thoracic vascular assessments, and preoperative scans were excluded. Additionally, scans in which the coronary CT angiogram was canceled due to very high coronary artery calcium (CAC) score were excluded. The remaining cohort was organized by maximum per-patient coronary stenosis or CAD-RADS as applicable. CCT, cardiac computed tomography; EP, electrophysiology; HF, heart failure; CMP, cardiomyopathy; CAC, coronary artery calcium; CAD, coronary artery disease; CCTA, coronary computed tomography angiography; CAD-RADS, coronary artery disease reporting & data system

We queried the picture archiving and communication system (PACS) for all CCTAs performed for clinical purposes between May 1, 2015 and June 30, 2017. CCTAs over a 12-month period prior to local CAD-RADS adoption were included in the non-standardized reporting (NSR) cohort. CCTAs performed in the 12 months following CAD-RADS adoption were included in the CAD-RADS cohort. A two-month window was excluded between the two cohorts to account for variable rates of adoption among imaging providers. CCTAs performed for reasons other than the evaluation of coronary atherosclerosis were excluded (Figure 1). Baseline demographic data were obtained through chart abstraction of a comprehensive electronic medical record (EMR) system. Hypertension was defined as a diagnosis of hypertension or utilization of an antihypertensive medication prior to CCTA. Hyperlipidemia was defined as a fasting LDL >190 mg/dL, a diagnosis of dyslipidemia in the EMR, or treatment with lipid-lowering medication. Diabetes mellitus was defined as a hemoglobin A1c ≥6.5% or prescription of anti-hyperglycemic medications. Patients with prior myocardial infarction (MI), percutaneous coronary intervention (PCI), or coronary artery bypass graft (CABG) surgery prior to CCTA were excluded for this analysis. Active smoking was adjudicated based on smoking status as documented in the provider encounter during which the CCTA was ordered. The medical specialty of the ordering provider was also abstracted, along with the rate of downstream testing and cardiology referrals following CCTA.

Coronary CT angiography 

All CCTAs were performed in accordance with the published guidelines utilizing either a 128-slice dual-source CT scanner (Definition Flash CT, Siemens Healthcare) or a 320-slice CT scanner (Aquilion ONE Vision, Toshiba Medical Systems) [14]. Coronary artery calcium (CAC) acquisitions were performed on all patients ≥40 years of age at the discretion of the CT technologist and imaging provider and interpreted utilizing the Agatston method [15]. Image interpretation was performed on a 3D workstation (Vital Images, Inc) by a level II or III cardiac imaging cardiologist or radiologist [16-17]. During the study period, all CCTAs were interpreted by the same cohort of cardiologists with the exception of one additional cardiologist prior to the adoption of CAD-RADS.

Coronary CT angiography reporting

CCTA scans performed prior to CAD-RADS implementation were interpreted and reported in accordance with previously published societal guidelines [18]. A normal scan was free of plaque or stenosis with a CAC score of zero (if performed). Non-obstructive CAD was defined as maximal per-patient stenosis between 1% and 49% or any CAC, and obstructive CAD was defined as a maximal per-patient stenosis ≥50%. The inclusion of recommendations from the cardiac imaging specialist in the report was at the discretion of the interpreting provider and was performed rarely in the NSR cohort. The imaging report format in the CAD-RADS cohort was modeled after the recommended template included in the published document (Table 1) [13]. Thus, a similar per-patient conclusion statement was reported: normal (no plaque/CAC zero/CAD-RADS 0), minimal (<25% stenosis/CAD-RADS 1) or mild (25% to 49% stenosis/CAD-RADS 2) non-obstructive CAD, and moderate (50% to 69% stenosis/CAD-RADS 3) or severe (70% to 100% stenosis/CAD-RADS 4/5) obstructive CAD. Additionally, the corresponding recommendation for stable chest pain was included in the report for all patients. In cases where the CCTA was performed for acute chest pain, the corresponding CAD-RADS statement pertaining to the likelihood of acute coronary syndrome (ACS) based on the CCTA findings was also included.

**Table 1:** CAD-RADS reporting recommendations LM, left main coronary artery; ACS, acute coronary syndrome; ICA, invasive coronary angiography; ECG, electrocardiogram; CAD, coronary artery disease; CAD-RADS, coronary artery disease-reporting & data system

CAD-RADS ™		0	1	2	3	4	5	N
Maximal Stenosis		0% (No plaque or stenosis)	1% to 24% (Minimal stenosis or plaque with no stenosis)	25-49%	50-69%	A: 70% to 99% B: LM >50% or 3v obstructive disease	100%	Non-diagnostic Study
Acute Chest Pain	Conclusion	ACS Highly Unlikely		ACS Unlikely	ACS Possible	ACS Likely	ACS Very Likely	Cannot exclude ACS
	Recommended Intervention	None	None	None unless high clinical suspicion or high-risk plaque features, then consider hospital admission with Cardiology consultation	Consider hospital admission with Cardiology consultation and/or ICA	Consider hospital admission with Cardiology consultation. Further evaluation with ICA and revascularization as appropriate.	Consider expedited ICA on a timely basis and revascularization if appropriate if acute occlusion.	Additional or alternative evaluation for ACS is needed
	Management	No further evaluation of ACS is required. Consider other etiologies.	Consider the evaluation of non-ACS etiology, if normal troponin and no ECG changes. Consider referral for outpatient follow-up for preventive therapy and risk factor modification.	Consider the evaluation of non-ACS etiology, if normal troponin and no ECG changes. Consider referral for outpatient follow-up for preventive therapy and risk factor modification.	Recommendation for anti-ischemic and preventive management should be considered as well as risk factor modification. Other treatments should be considered if the presence of hemodynamically significant lesion.	Recommendation for anti-ischemic and preventive management should be considered as well as risk factor modification.	Recommendation for anti-ischemic and preventive management should be considered as well as risk factor modifications.	
Stable Chest Pain	Conclusion	Absence of CAD	Minimal non-obstructive CAD	Mild non-obstructive CAD	Moderate stenosis	Severe stenosis	Total coronary occlusion	Cannot Exclude Obstructive CAD
	Recommended Intervention	None	None	None	Consider functional assessment	A: Consider ICA or functional assessment B: ICA is recommended	Consider ICA and/or viability assessment	Additional or alternative evaluation may be needed
	Management	Reassurance. Consider non-atherosclerotic causes of chest pain.	Consider non-atherosclerotic causes of chest pain. Consider preventive therapy and risk factor modification.	Consider non-atherosclerotic causes of chest pain. Consider preventive therapy and risk factor modification, particularly for patients with non-obstructive plaque in multiple segments.	Consider symptom-guided anti-ischemic and preventive pharmacotherapy as well as risk factor modification per guideline-directed care. Other treatments should be considered per guideline-directed care.	Consider symptom-guided anti-ischemic and preventive pharmacotherapy as well as risk factor modification per guideline-directed care. Other treatments (including options of revascularization) should be considered per guideline-directed care.	Consider symptom-guided anti-ischemic and preventive pharmacotherapy as well as risk factors modification per guideline-directed care. Other treatments (including options of revascularization) should be considered per guideline-directed care.	

Downstream testing & subspecialty referral

Given the short duration of follow-up time from the date of CCTA in both cohorts, any downstream ischemic testing was considered related to CCTA. Exercise treadmill stress, stress echocardiography (exercise or dobutamine), vasodilator stress cardiac magnetic resonance, and single-photon emission computed tomography (SPECT) myocardial perfusion imaging (exercise or vasodilator) were considered downstream functional testing. Invasive coronary angiography (ICA) or repeat CCTA was considered downstream anatomic testing. The initial telephone or in-person provider note following CCTA was reviewed. Cardiology referrals placed during this visit were considered related to CCTA.

Cost analysis

Cost analysis was based on direct patient charges utilizing the Center for Medicare & Medicaid Services (CMS) outpatient prospective payment system (OPPS) final rule for 2018. Non-invasive testing costs were based on the following CPT codes: 77574, 78452, 93351, 75563, and 93015. ICA with left heart catheterization (LHC) was utilized when invasive fractional flow reserve (FFR) and percutaneous coronary intervention (PCI) was deferred. The incremental cost for the performance of invasive FFR and PCI was included when applicable.

Statistical analysis

Statistical analysis was performed using IBM SPSS version 22.0 (IBM, Armonk, NY) and JMP version 13.3 (SAS Corp, Cary, NC). Parametrically distributed continuous variables are reported as means with standard deviation and nonparametric data are reported as medians with interquartile range. Comparison between continuous variables was performed utilizing a 2-tailed Fisher’s exact test or a Wilcoxon Rank-sum test, as appropriate. A Bonferroni correction was applied when appropriate.

## Results

Population

The CAD-RADS reporting template was utilized in 83.7% of CCTAs performed during the CAD-RADS adoption period. In asymptomatic patients undergoing CCTA, NSR was more frequently used (*p *= 0.033). The ordering provider was more commonly a primary care provider in the NSR cohort (*p *= 0.047), whereas CAD-RADS was more commonly utilized in studies ordered by a cardiology provider (*p *= 0.003). Baseline cardiovascular risk factors were well balanced between the groups (Table 2).

**Table 2:** Baseline demographic data NSR, non-standardized reporting; ATCP, atypical chest pain; CP, chest pain; ED, emergency department; CT, computed tomography; HTN, hypertension; CHD, coronary heart disease; CAD, coronary artery disease; CAC, coronary artery calcium; IQR, interquartile range; ASA, aspirin; CAD-RADS, coronary artery disease-reporting & data system

	CAD-RADS Cohort (n = 751)	NSR Cohort (n = 1045)	p-value
Study Indications			
ATCP	612 (81.5%)	823 (78.8%)	0.159
Dyspnea	20 (2.7%)	34 (3.3%)	0.465
Syncope	22 (2.9%)	25 (2.4%)	0.656
Abnormal Stress Test	11 (1.5%)	29 (2.8%)	0.067
Acute CP/ED CT	32 (4.3%)	28 (2.7%)	0.064
Asymptomatic	54 (7.2%)	106 (10.1%)	0.033
Age	50±12	49±13	0.133
Male gender	446 (59.4%)	577 (55.2%)	0.082
Active Smokers	94 (12.5%)	165 (15.8%)	0.057
Diabetes	111 (14.8%)	137 (13.1%)	0.332
HTN	364 (48.5%)	499 (47.8%)	0.774
Dyslipidemia	333 (44.3%)	468 (44.8%)	0.885
Known CHD	53 (7.1%)	77 (7.4%)	0.854
CCTA CAD burden			
Normal	360 (47.9%)	595 (56.9%)	<0.001
Non-obstructive CAD	247 (32.9%)	334 (32.0%)	0.689
Obstructive CAD	29 (3.9%)	51 (4.9%)	0.313
Non-diagnostic Scan/Segment	73 (9.7%)	44 (4.2%)	<0.001
Any CAD	276 (36.8%)	385 (36.8%)	1.000
CAC score (median, IQR_25,75_)	0 (0, 24.5)	0 (0, 22)	0.114
CAC percentile	76.7±18.3	78.2±18.0	0.288
Incidence CAC > 75^th^ percentile	177 (26.0%)	213 (22.4%)	0.099
Baseline Medical Therapies			
Statin	277 (36.9%)	358 (34.3%)	0.271
Non-statin	34 (4.5%)	55 (5.3%)	0.510
ASA	267 (35.6%)	352 (33.7%)	0.421
Antihypertensive	342 (45.5%)	482 (46.2%)	0.810

Downstream testing

**Figure 2:**
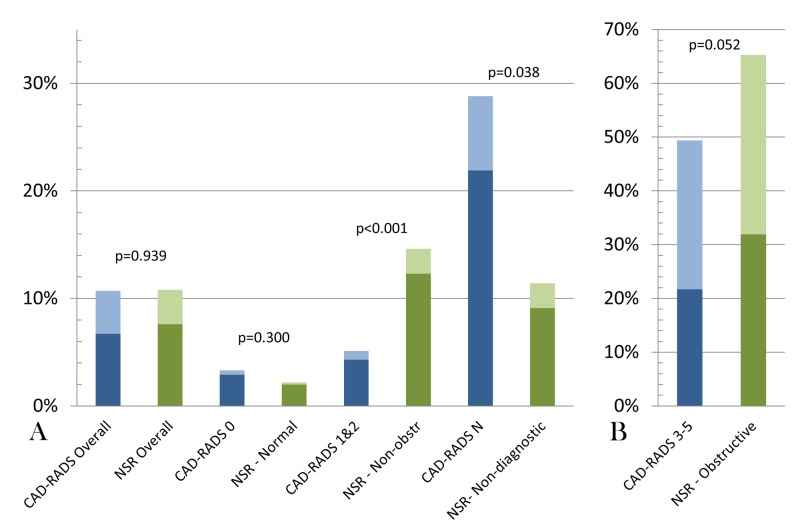
Downstream testing rates before and after the adoption of the CAD-RADS standardized reporting template Downstream testing in the CAD-RADS (blue) compared with NSR (green) cohorts. The dark color represents functional testing and the lighter color represents coronary anatomy testing. There were no differences in functional or anatomic testing rates between CAD-RADS and non-standardized reporting (NSR) cohorts within each stenosis severity category. Functional testing was far more common in all categories with the exception of obstructive CAD/CAD-RADS 3-5. Panel A: Graphical representation of CAD-RADS and NSR cohorts overall and with non-obstructive/non-diagnostic scans; Panel B: Graphical representation of CAD-RADS and NSR cohorts with obstructive coronary artery disease CAD-RADS, coronary artery disease-reporting & data system; NSR, non-standardized reporting; Non-obstr, non-obstructive; N, non-diagnostic

Referrals for additional downstream testing (Figure 2) were similar between the CAD-RADS and NSR cohorts (10.7% vs 10.8%, *p *= 0.939). The rates of referral for downstream functional testing and anatomic testing were also similar (*p *= 0.276 & 0.226, respectively). Functional testing was more commonly performed in all stenosis severity categories with the exception of obstructive CAD/CAD-RADS 3-5 (Figure 2). In patients with no CAD, downstream testing referrals were rare (3.3% vs 2.2%, *p *= 0.300), driven almost entirely by functional testing (88.0% vs 92.3%, *p *= 0.593). Among patients with non-obstructive CAD, referral for downstream testing (Figure 2) was reduced in CAD-RADS 1 & 2 patients compared to NSR (5.1% vs 14.4%, *p *< 0.001). Downstream testing was numerically lower in CAD-RADS 3 through five patients compared with obstructive CAD in the NSR cohort (49.4% vs 65.3%, *p *= 0.052). These were offset by a higher incidence of CAD-RADS N scans compared to non-diagnostic scans within the NSR cohort (9.7% vs 4.2%, *p *< 0.001). The imaging report was significantly more likely to include recommendation for downstream testing in the CAD-RADS cohort compared to NSR (60% vs. 9.3% *p *< 0.001). This was associated with an increased observed incidence of downstream testing (28.8% vs 11.4%, *p *= 0.038). In both the CAD-RADS and NSR cohorts, non-diagnostic scans were attributed to poor visualization of epicardial vessels, branch vessels or both at similar rates (54.8% vs. 50%; 2.7% vs. 11.4%; 42.5% vs. 38.6%, respectively), When excluding patients with normal or non-diagnostic CCTAs, downstream testing was more commonly pursued in the NSR cohort (23.4% vs 15.5%, *p *= 0.009).

Invasive coronary angiography

Referrals for invasive coronary angiography (ICA) were low in both groups overall (3.5% in CAD-RADS and 3.2% in NSR, *p *= 0.726). Among patients referred for ICA, the incidence of confirmed obstructive CAD was high in both groups (61.5% vs 69.7%, *p *= 0.585). Among patients with obstructive CAD, referrals for ICA were similar between the groups (56.1% vs 51.1%, *p *= 0.638), as was the rate of obstructive CAD on ICA (*p *= 0.193). Despite lower downstream testing rates in CAD-RADS patients after excluding those with no CAD and non-diagnostic scans, there was no difference in the incidence of confirmed obstructive CAD on ICA (74.2% vs 66.7%, *p *= 0.565).

Referrals to cardiology

After excluding CCTAs ordered by cardiology providers, the rate of cardiology referral by primary care providers (PCPs) were similar when comparing the CAD-RADS to NSR cohorts (12.2% vs 15.8%, *p *= 0.197). Cardiology referrals, irrespective of reporting template utilized, were more common among patients with any CAD on CCTA (21.8% vs 9.4%, *p *< 0.001). In patients with no CAD, cardiology referrals were low and did not differ between the reporting template groups (*p *= 0.431). In patients with obstructive CAD, cardiology referral rates were higher but again did not differ between the reporting template cohorts (*p *= 0.865). Conversely, significantly higher rates of cardiology referrals from PCPs were observed for non-obstructive CAD in the NSR cohort compared with CAD-RADS 1 & 2 patients (20.5% vs 8.6%, *p *= 0.021). Referrals to a cardiology provider were similar between reporting cohorts in patients with non-diagnostic scans/CAD-RADS N (16.0% vs 15.8%, *p *= 0.984). After excluding patients with normal or non-diagnostic CCTAs, no difference was observed in the PCP referral rates to cardiology between the reporting cohorts (CAD-RADS: 16.7% vs NSR: 25.0%, *p *= 0.107).

Cost analysis

**Figure 3:**
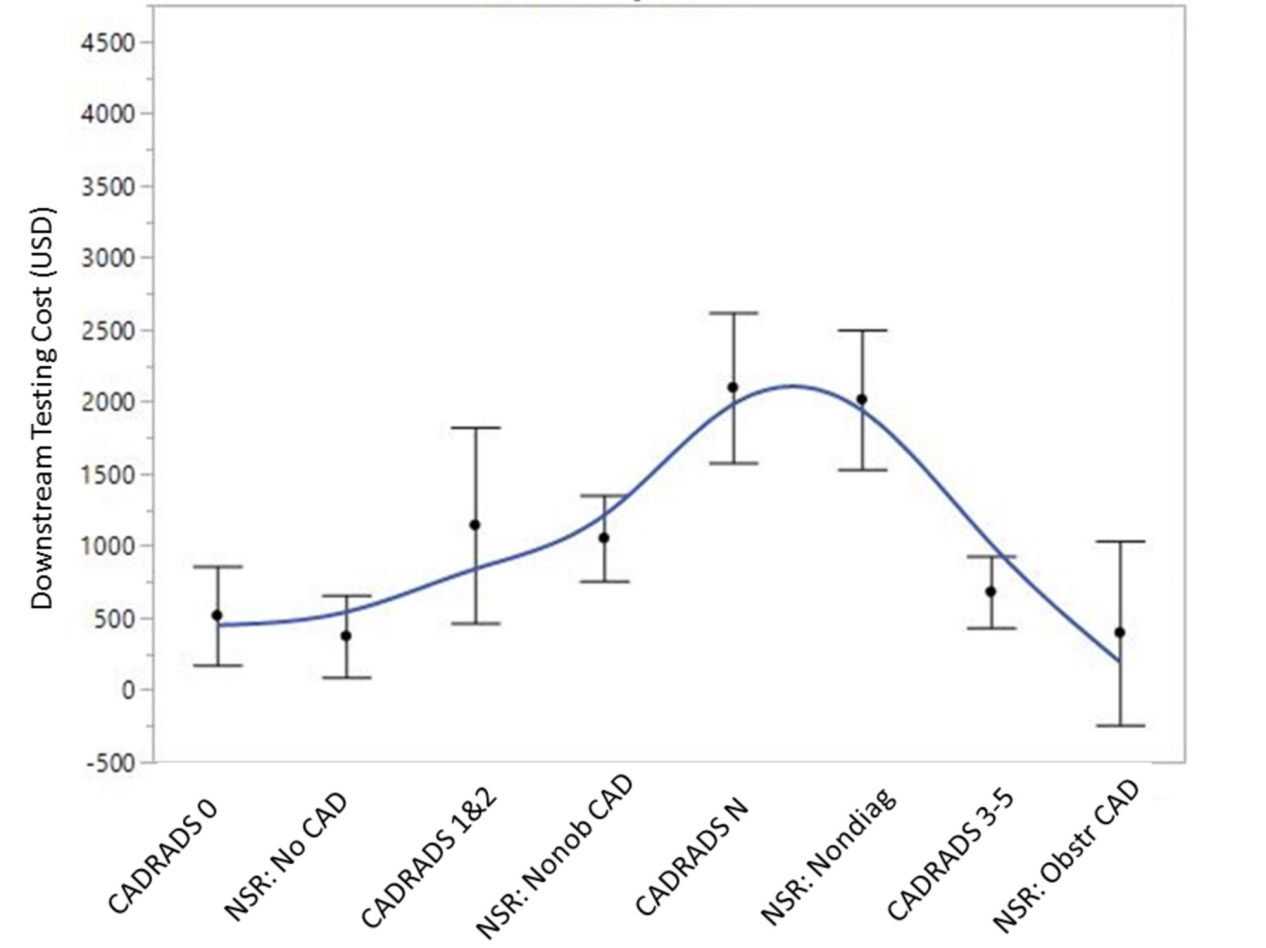
Comparison of median downstream testing costs between the CAD-RADS and non-standardized reporting cohorts Spline curve with error bars depicting the median downstream testing costs based on per-patient maximum coronary stenosis between the CAD-RADS and NSR cohorts. P = NS for all between-group comparisons. Downstream testing costs were higher for CAD-RADS N and NSR nondiagnostic scans compared to patients with no CAD/CAD-RADS 0 and obstructive CAD/CAD-RADS 3-5 (*p *< 0.05). CAD-RADS, coronary artery disease-reporting & data system; Nonob CAD, non-obstructive coronary artery disease; Nondiag, non-diagnostic; Obstr CAD, obstructive coronary artery disease; NSR, non-standardized reporting; USD, United States Dollar

Median downstream total testing costs (Figure 3) were similar when CAD-RADS or NSR templates were utilized ($1248; $526-$7750 vs $1248; $825-$7750, *p *= 0.554). When incorporating cardiology referral costs based on a 99203 encounter with any downstream testing costs, there was no difference between the groups (*p *= 0.598). When comparing per-patient maximum stenosis severity categories between the groups, there was no difference in median total testing costs or testing costs plus cardiology referral costs.

## Discussion

Utilization of the CAD-RADS reporting template in a single-center, closed-referral military health system was not associated with changes in cardiology referral rates among PCPs, downstream testing, or overall testing costs. However, a reduction in downstream testing and cardiology referral were observed among patients with non-obstructive CAD by CAD-RADS (CAD-RADS 1 & 2) compared with NSR. This difference was offset by higher rates of non-diagnostic scans (CAD-RADS N) in the CAD-RADS cohort that resulted in higher downstream testing rates compared with the NSR cohort. We suspect the higher rate of non-diagnostic scans observed in the CAD-RADS cohort was secondary to the explicit criteria for non-diagnostic scans delineated in the CAD-RADS reporting template. This suggests that the adoption of the CAD-RADS reporting template was associated with increased resource utilization in the non-diagnostic cohort which negated the decrement in downstream testing & cardiology referral that was observed in the CAD-RADS 1&2 cohort. There was no significant difference in vessel involvement (epicardial vs. branch vessel) that was attributed to the non-diagnostic scans in both cohorts which suggests in the NSR cohort the decision to pursue further downstream testing was a result of the ordering providers interpretation of the data presented. Given these findings, the adoption of the CAD-RADS template with the inclusion of recommendations for further downstream testing in the event of a non-diagnostic scan likely contributed to the observed increase in downstream testing in that cohort. Excluding nondiagnostic scans/CAD-RADS N, observed downstream testing, and cardiology referrals were reduced when CAD-RADS was utilized without a reduction in the detection of obstructive CAD on ICA.

Large prospective trials and registry data have described the effect that CCTA has on downstream testing, ICA and revascularization. All of these studies, with the exception of the Scottish Computed Tomography of the Heart (SCOT HEART) trial, reported increased rates of ICA and revascularization [1,19-20]. SCOT HEART notably did report higher rates of ICA and revascularization in the CCTA group initially, but overall rates were similar at five years. CCTA utilizing non-standardized reporting systems have been evaluated previously with prior analyses concluding that CCTA is likely a cost-effective alternative to functional testing [21]. In fact, an independent economic analysis performed for the NICE guidelines concluded that CCTA provided the lowest cost per correct diagnosis [10]. Our analysis evaluated the association of CAD-RADS reporting system adoption on the observed rate of downstream testing, cardiology referral rates, and invasive coronary angiography. In comparison to prior studies in which these outcomes were reported in CCTA compared to functional testing, we observed a lower rate of utilization of downstream testing in patients with non-obstructive disease offset by higher utilization in patients with nondiagnostic studies. While this did not change the overall cost or downstream utilization rate, it may represent a reallocation of resources toward patients must likely benefit from further testing. Importantly, this reduced testing and cardiology referral rate did not significantly change the incidence of obstructive CAD on ICA in the CAD-RADS cohort. 

The role of the imaging report is vital to communicating pertinent test findings to the referring provider so that appropriate interventions can be pursued. The primary means by which a diagnostic imaging test impacts patient outcomes is through effective communication of important findings to the treating provider. There are several examples of standardized reporting systems positively impacting patient outcomes, namely the Breast Imaging Reporting and Data System (BI-RADS) and Liver Imaging Reporting and Data System (LI-RADS) systems for reporting breast and liver imaging findings, respectively [22-23]. These standardized reporting systems have been associated with a reduction in the incidence of reporting errors and miscommunication, likely as a result of a reduction in interpretation variability [24]. Conversely, a lack of standardized reporting can result in misinterpretation of patient risk and the potential for under treatment. In the study of Myocardial Perfusion and Coronary Anatomy Imaging Roles in Coronary Artery Disease (SPARC), roughly 50% of patients with high-risk non-invasive cardiac imaging findings were not referred for ICA. Additionally, up to 30% were not prescribed aspirin and up to 25% were not prescribed lipid-lowering agents [12]. Our analysis seems to support that implementation of CAD-RADS standardized reporting effectively directs providers to pursue testing appropriately in patients with the highest risk findings while empowering them to defer testing when non-obstructive CAD is present. Further evidence for improved communication associated with the adoption of the CAD-RADS template was the observed increase in an appropriately ordered downstream testing and cardiology referrals in patients who have non-diagnostic CCTAs in the CAD-RADS cohort compared to NSR which likely represents a decrease in interpretation variability.

Conclusions from this data are limited by the fact that it was derived from a single-center, closed-loop, single-payer military treatment facility, thus the results may not be applicable to other healthcare systems. Our data were obtained via retrospective chart review which limits the ability to establish causality. A higher incidence of non-diagnostic CCTA (CAD-RADS N) was observed in the CAD-RADS cohort when compared to NSR. A non-diagnostic rate approaching 10% in the CAD-RADS cohort is high when compared to published CAD-RADS data which is approximately 4% [25]. The increased non-diagnostic rate in our population may be explained by a higher proportion of early career CT readers at our institution, patient parameters, and strict application of the CAD-RADS criteria for non-diagnostic scans which specifies that a study is non-diagnostic if not all vessel segments >1.5 mm in diameter can be interpreted with confidence [13]. Finally, there were considerable differences in follow-up between the reporting cohorts precluding any analysis of cardiovascular outcomes based on reporting template utilized.

## Conclusions

Adoption of the CAD-RADS reporting template was associated with a reduction in downstream testing and cardiology referral rates among non-obstructive CAD (CAD-RADS 1 & 2) patients. Thus, CAD-RADS may impact downstream testing in patients in whom further testing can typically be deferred. 
